# Measuring health-related quality of life in infants and toddlers: conceptual challenges and proposed recommendations

**DOI:** 10.1007/s11136-026-04199-8

**Published:** 2026-04-01

**Authors:** Philip A. Powell, Fanni Rencz, Jill Carlton, Simone Schieskow, Tessa Peasgood, Brendan Mulhern, Aureliano Finch, Mike Herdman, Janine Verstraete

**Affiliations:** 1 School of Medicine and Population Health, University of Sheffield, Sheffield, UK; 2 EuroQol Research Foundation, Rotterdam, The Netherlands; 3 Department of Health Policy, Corvinus University of Budapest, Budapest, Hungary; 4 Department of Health Economics and Health Care Management, Faculty of Health Science, Bielefeld University, Bielefeld, Germany; 5 Centre for Health Economics Research and Evaluation, University of Technology Sydney, Sydney, Australia; 6 Saw Swee Hock School of Public Health, National University of Singapore, Singapore, Singapore; 7 Department of Paediatrics and Child Health, Faculty of Health Sciences, University of Cape Town, Cape Town, South Africa

**Keywords:** Early childhood, Health-related quality of life, Health technology assessment, Observer-reported outcomes, Preference-weighted measures, Proxy reporting

## Abstract

**Purpose:**

Measuring health-related quality of life (HRQoL) in the very young (i.e., infants and toddlers, aged 0–47 months) presents unique conceptual and methodological challenges. As infants and toddlers cannot reliably self-report their HRQoL, observer inference is necessary. This raises questions about which concepts should be included; how to manage proxy reporting; and how to capture genuine variations in HRQoL, not changes in development or assessment. This commentary outlines six key challenges in measuring infant and toddler HRQoL and details eight proposed recommendations for HRQoL researchers.

**Methods:**

The piece draws on insights from the EuroQol Toddler and Infant Populations (EQ-TIPS) project, aimed to develop a generic preference-weighted measure (PWM) of HRQoL for infants and children, but has broader applicability to HRQoL measurement in the very young. Key issues addressed include: (i) what concepts to measure; (ii) how to identify which concepts matter to very young children; (iii) managing proxy reporting and reducing bias; (iv) accounting for rapid developmental changes; (v) managing continuity across life-course instruments; and (vi) separating child HRQoL from family spillover effects.

**Results:**

Proposed recommendations include greater consensus on a core HRQoL model; prioritising primary caregiver perspectives; justifying and operationalising observable aspects of subjective HRQoL; careful design to capture genuine HRQoL, not developmental change or caregiver spillover; and developing measurement systems to prioritise age-based sensitivity or comparability across time.

**Conclusion:**

These recommendations offer a foundation for future consensus-building and research to refine and harmonise best practices in infant and toddler HRQoL measurement, particularly for use in health technology assessment.

## Introduction

Alongside survival, health-related quality of life (HRQoL) is a critical outcome in patient welfare, clinical trials, and the cost-effectiveness analyses of healthcare interventions [1]. While definitions of HRQoL vary [2], they typically encompass health-related impacts in at least three areas – physical, psychological, and social functioning – and emphasise inherent subjectivity. Accordingly, outcome measures are designed, wherever possible, to capture *self-reported* HRQoL. Preference-weighted measures (PWMs) are further adapted to be scored with preference weights for each dimension of HRQoL. When weighted relative to preferences for survival, this allows for the calculation of quality-adjusted life years (QALYs) for use in economic evaluation as part of health technology assessment (HTA). HRQoL and QALYs are not restricted to adults, and there are many measures of HRQoL developed for use in childhood [3, 4]. However, when it comes to measuring HRQoL in the very young, that is infants (i.e., 0–11 months) and toddlers (i.e., 12–47 months), there are several unique methodological challenges [5]. Such challenges are particularly pertinent in the context of HTA, where HRQoL data are used to help assess the cost-effectiveness of healthcare interventions for infants and toddlers [6]. Ensuring that HRQoL data used for infants and toddlers is both valid and reliable is important and has practical implications for healthcare decision-making. In this commentary we outline the latest thinking on measuring HRQoL in infants and toddlers. In doing so, we draw on the collective experience and lessons learned as part of the EuroQol Toddler and Infant Populations (EQ-TIPS) project, designed to develop a generic HRQoL PWM for infants and toddlers [7–12]. The commentary builds on a previous position piece published in 2019 addressing the measurement of HRQoL in very young children [5], and complements a recent paper examining issues in health state valuation for infants and toddlers [13]. In this commentary we articulate six key challenges involved in measuring infant and toddler HRQoL and detail eight proposed recommendations for HRQoL researchers to consider to begin addressing these challenges.

## The need for HRQoL measurement in infants and toddlers

Children aged 0–47 months are estimated to represent 7.8% of the world’s population [14] and accounted for an estimated 25% of the global disease burden in 2019 [15]. Targeted interventions under the World Health Organisation’s (WHO) Sustainable Development Goals (SDGs) have translated into a decrease in the burden of communicable disease in this age group over the last 30 years. As more countries meet the targets for the SDGs their focus is shifting to managing non-communicable diseases e.g., congenital conditions, orphan diseases and genomic medicine with a focus on improving health, increasing life years and improving HRQoL. Thus, there is a resultant demand for sensitive measures of health and HRQoL in this very young age group [6]. This need extends to PWMs suitable for infants and toddlers. A review of the Pediatric Economic Database Evaluation (PEDE) from 1997 to 2009 showed that 29.2% of submissions for Cost Utility Analysis (CUA) were for infants up to one year of age [16]. However, submissions for children younger than five years to the National Institute for Health and Care Excellence (NICE) in the UK have mostly used generic HRQoL measures and values designed for adults [6], with a similar situation for child submissions in Australia [17].

A reliance on measures and values designed for adults presents significant challenges. For example, adult instruments typically emphasise domains such as independence, self-care, and psychological functioning, which may not be developmentally appropriate or readily observable in infants and toddlers. This, combined with the omission of early-life concepts, such as feeding, sleeping, and play, can result in limited content validity for the very young [18]. Further, adult-based value sets – reflecting concepts relevant to and/or from the perspective of being important to adults themselves – may not be transferable to infants and toddlers, leading to biased utility estimates [13]. This prompts a need for a dedicated PWM that fits the needs of very young children.

The EQ-TIPS was funded by the EuroQol Research Foundation to meet the abovementioned need. The experimental measure is an advancement and refinement of the existing Toddler and Infant (TANDI) generic HRQoL measure [7, 8] and is recommended for children aged 0–47 months. EuroQol experimental measures are tools which are still under development and may change as work proceeds. Experimental version 3.0 of the EQ-TIPS includes seven items of HRQoL, namely: movement, eating or drinking, sleep, pain, managing emotions, interacting with others and play [13, 19]. Both three-level (EQ-TIPS-3 L) and five-level (EQ-TIPS-5 L) response variations are available. A proxy respondent, typically the parent, is asked to indicate the child’s HRQoL ‘Today’. The items were developed from scratch but many of the characteristics of the EQ-5D-Y [20], proxy version 1, including the layout, font, instructions, severity level qualifiers, EQ VAS and recall period [21], were retained [7]. The EQ-TIPS was initially developed and tested in South Africa with promising psychometric results [9–11, 22]. Version 2.0 of the measure has also performed well in other countries [23–26]. Further work is informing ongoing development and assessing version 3.0 of the measure in diverse language groups and sociocultural settings, as well as the methods for its valuation [13].

As part of six years of multi-country research, six conceptual challenges for measuring infant and toddler HRQoL were identified, each requiring a proposed stance or recommendation (see Table 1). These are: (i) what concepts to measure; (ii) how to identify which concepts matter; (iii) proxy reporting; (iv) rapid developmental change; (v) transitioning between instruments; and (vi) spillover effects.

**Table 1:** Challenges and recommendations for measuring HRQoL in infants and toddlers

Challenge	Proposed recommendation(s)
1. What concepts to measure HRQoL priorities vary with age, making universal models unsuitable across the lifespan. Historically, limited HRQoL models/measures for children under 5 years. Several measures now available, but heterogeneity in HRQoL concepts captured. Lack of core HRQoL conceptual model limits comparability and synthesis across studies and adds to uncertainty in HTA. Emphasis on observable states aligns with FDA guidance but risks overlooking subjective experiences.	1. Need for greater consensus on a core conceptual model of HRQoL for infants and toddlers. Aim to achieve consensus from multiple viewpoints, including primary caregivers, developmental and clinical experts, and HRQoL specialists. 2. More subjective aspects can be considered for inclusion where evidence-based observable correlate(s) exist (e.g., crying for pain/distress). The inclusion of these constructs should be operationalised for measurement via an observer (i.e., by providing reliable behavioural correlates).
2. How to identify which concepts matter? Developers of infant and toddler HRQoL measures have used different techniques to decide which concepts to include. Most were adapted from older populations with limited or secondary parent-caregiver involvement. There is a need to prioritise primary caregiver input through established qualitative methods and peer-to-peer collaborative involvement. Secondary input and guidance from professionals likely to be beneficial.	3. Conceptual/measurement models of infant and toddler HRQoL should prioritise the perspectives of primary caregivers through established qualitative methods. Ideally, pluralistic views will be incorporated, but greater weight should be afforded to people with the closest link to the target population and/or investment in their welfare. Research activities should be complemented with dedicated peer-to-peer advisory work with people with lived experience.
3. Proxy reporting Infants and toddlers cannot self-report. Different proxy versions are possible (i.e., based on proxy’s own perception or perception of how they think the child would respond). Subjectivity cannot be eliminated and varies by proxy’s characteristics. Proxy could vary (e.g. parent, HCP, childminder).	4. Due to their intimate and close link to their infant/toddler, the primary caregiver should act as the preferred proxy by default. Although this may vary by sociocultural context or study methodology. 5. An observer-based approach to proxy report is essential for infants and toddlers and is theoretically consistent with an ObsRO, whereby the observer should answer on behalf of the child (rather than inferring how the child would respond).
4. Rapid developmental change Rapid and uneven developmental changes in early childhood complicate HRQoL assessment. Rather than capturing normal developmental variation, HRQoL instruments should focus on health-related aspects (and change in HRQoL only).	6. Special care needs to be taken when designing items for measuring HRQoL in infants and toddlers to account for rapid developmental change. Ensure items are constructed to best detect changes in HRQoL and not expected changes in development for the intended age range of the measure.
5. Transitioning between instruments Lifespan HRQoL measurement aims for continuity, but changes in instruments, reporting methods (e.g., proxy vs. self-report), or value sets can introduce discontinuities unrelated to true health changes. Age-specific tools increase sensitivity but risk reduced comparability over time, especially during transitions.	7. Consider the importance of continuity in HRQoL assessment as part of the measurement model and valuation strategy in young children. If the intended context of use prioritises consistency over time (e.g., comparing across/between age groups and/or consistent QALY generation) then develop measures that maximise commonality across age groups. Otherwise, age-based sensitivity could be prioritised (e.g., through age-specific measures).
6. Spillover effects Separating infant/toddler HRQoL from caregiver and family well-being is challenging due to high dependency and bidirectional spillover effects, especially in chronic illness. This overlap risks double counting in evaluations if caregiver impacts are measured separately but also reflected in child-focused tools.	8. HRQoL measures for infants and toddlers should be designed to minimise capturing ‘spillover’ HRQoL of caregivers or wider family, which can be assessed using complementary, independent instruments.

FDA = U.S. Food and Drug Administration. HCP = Healthcare professional. HRQoL = Health-related quality of life. ObsRO = observer-reported outcome measure. QALY = quality-adjusted life year

## Challenges and proposed recommendations

### Challenge 1: what concepts to measure

While commonalities are observed in aspects of HRQoL that matter to different people, known developmental state-related variation exists [27]. Different dimensions of HRQoL become more or less relevant as people age [28]. For example, parent-relational aspects may be more prominent for younger than older children, or adults [29]. Further, the same HRQoL dimension may be relevant across the entire lifespan (e.g., mobility) but vary in how it is defined conceptually and assessed. For this reason, it is unlikely that a universal model of HRQoL (i.e., featuring the same operationalised dimensions), would generalise well across the lifespan, including to infants and toddlers.

Historically, there was a lack of adequate HRQoL measures and associated conceptual frameworks designed specifically for use in children under 5 years old [28, 30]. However, several instruments have now been developed for this purpose, including but not limited to the Infant and Toddler Quality of Life Questionnaire (ITQOL; 2 months – 5 years) [31, 32], TNO-AZL Pre-School Children Quality of Life Questionnaire (TAPQOL; 1–5 years) [33], and the Pediatric Quality of Life Inventory (PedsQL) scales for infants (1–24 months) and toddlers (2–4 years) [34–37]. As well as the EQ-TIPS, PWMs for very young children also exist, including the Health Utilities Pre-School (HuPS, 2–4 years) [38, 39] and the Infant Health-Related Quality of Life Instrument (IQI; 0–12 months) [40, 41]. The Child Health Utility 9D (CHU9D) was recently adapted for use in 2–5-year-olds [42, 43], and two health state classification systems for generating utilities for the PedsQL, for children and adolescents 2–18 years, are available, with value set estimation underway [44, 45].

Across these instruments there is heterogeneity in the HRQoL concepts captured. Existing measures cover a breadth of domains, such as physical abilities, health problems, and socio-emotional functioning. However, there are differences in the concepts included in each instrument and how they are operationalised. For example, TAPQOL includes wellbeing related to physical health complaints (e.g., stomach and lung problems) [33]. PedsQL infant and toddler scales include items on specific emotions, such as worry, sadness, and anger [34, 36]. EQ-TIPS was designed around readily observable behaviours [18], resulting in a more targeted conceptual scope than some scales. As a result, instruments designed to assess HRQoL in the very young do not reflect a shared or unified core conceptualisation of HRQoL [46].

While some variation is expected based on developmental differences and the target age range, it also results from differences in underlying conceptual frameworks and instrument developers’ choices. As in other areas of the lifespan, instruments assessing infant and toddler HRQoL differ in the extent they prioritise aspects such as observable functioning, subjective experience states, and/or broader quality of life or familial impacts [46]. Conceptual inconsistency across measures has important implications for research and healthcare decision-making. It can limit comparability and synthesis across studies. Further, in HTA, differential assessments of HRQoL due to instrument choice (and resultant conceptual coverage) may result in different estimates of benefit and contribute to uncertainty in findings [47].

Given that the measurement of HRQoL for infants and toddlers requires observation, and following U.S. Food and Drug Administration (FDA) guidance, one may choose to restrict possible aspects of HRQoL to readily observable states only [48]. Indeed, this approach was adopted in the EQ-TIPS version 3.0, where items were operationalised to refer to observable states (e.g., for managing emotions: ‘the child can be comforted when upset’). However, other measures, such as PedsQL infant and toddler scales, include a range of subjective states (e.g., ‘worrying’, ‘afraid’, ‘angry’, ‘sad’), and their exclusion risks devaluing subjective experiential aspects in HRQoL measurement for very young children (vs. older children and adults that can *self-report*). There may be ambiguity in what is and is not fundamentally observable, which requires a judgement call (e.g., a subjective state of upset or distress can observably manifest through extended crying beyond developmental norms). Accordingly, researchers may need to justify the inclusion of any HRQoL factors that are important but appear subjective in their measurement model, clearly operationalising their behavioural correlates.

#### Proposed recommendation 1

There is a need for greater consensus around a core conceptual model of HRQoL for infants and toddlers. This conceptual framework should aim to achieve consensus from multiple viewpoints, including primary caregivers, developmental and clinical experts, and HRQoL specialists.

#### Proposed recommendation 2

More subjective aspects can be considered for inclusion in HRQoL measures for infants and toddlers where evidence-based observable correlate(s) exist (e.g., crying for pain/distress). The inclusion of any aspects that appear subjective should be operationalised for measurement via an observer (i.e., by providing reliable behavioural correlates).

### Challenge 2: how to identify which concepts matter

Developers of infant and toddler HRQoL measures used different techniques to decide which concepts were important to incorporate in their questionnaires. These included: adaptation of existing measurement models (typically first developed in older children and/or adults) [35–39, 42–45]; reviewing the literature and/or guidelines [7, 32, 33, 41]; input from paediatric healthcare professionals or experts [7, 33, 38, 40, 45]; and feedback from primary caregivers [7, 33, 40, 45]. For most of these measures the involvement of primary caregivers in determining content appeared secondary (i.e., items were generated based on the literature and/or expert input and then tested with parents, rather than parents inductively or collaboratively inputting into item generation, e.g. [40]). An exception is the recent neonatal and infant HRQoL (NIHRQOL; 0–1 years) model derived bottom-up from qualitative methods with caregivers and professionals [28]. There is, however, no infant measure related to this model currently available. Often, insufficient detail is provided to understand the development process transparently (e.g. [33]), which is a prevailing problem in paediatric outcomes research [49]. Further, where a measurement model of HRQoL is extrapolated from older to younger children there is a risk of raising problems related to applicability. The PedsQL for toddlers, for example, asks the parent how much of a problem their toddler has had with ‘lifting heavy things’.

There is therefore a need to ensure a consistent and agreed approach to deciding which concepts matter for HRQoL in very young children. Akin to the patients’ voice in PROM development for older children and adults, arguably – as representatives and guardians of their children and the closest to the target population – this should put *primary caregivers’ views* central to this process. Input from other key groups, such as developmental experts and/or paediatric healthcare professionals is also likely of benefit, but should be secondary to the primary caregiver.

Primary caregivers are likely to need some guidance in this process (i.e., setting the boundaries for what is and is not suitable within a PWM of HRQoL). This can be achieved through the involvement of HRQoL experts and appropriately tailored research methods. Best practice in concept elicitation involves qualitative methods to elicit and elucidate in detail concepts that matter for the target population [50]. This is typically followed by cognitive interviews to ensure the comprehensibility, relevance, and comprehensiveness of the HRQoL framework [51, 52]. Consensus activities, such as Delphi exercises can also be useful in moving towards a unified framework of HRQoL for infants and toddlers [53, 54]. While such research activities are an essential component of establishing a conceptual framework, they should be meaningfully complemented by peer-to-peer collaborative advisory work with primary caregivers and potentially other experts [55].

#### Proposed recommendation 3

The development of conceptual/measurement models of infant and toddler HRQoL should prioritise the perspectives of primary caregivers through established qualitative methods. Ideally, pluralistic views will be incorporated (e.g., developmental experts, healthcare professionals), but greater weight should be afforded to people with the closest link to the target population and/or investment in their welfare (i.e., parents or guardians). Research activities should be complemented with dedicated peer-to-peer advisory work with people with lived experience.

### Challenge 3: proxy reporting

It is accepted that toddlers and infants do not have sufficient cognitive or language skills to process, communicate, or self-report their health experience to others [56]. Accordingly, in assessing HRQoL we are reliant on someone to report on their behalf. The FDA and the International Society for Pharmacoeconomics and Outcomes Research (ISPOR) recommend that these respondents report young children’s health on observable behaviours, signs or events to reduce parental subjectivity and interpretation (e.g., measurement of parental concern) [48, 56]. Hence, the term Observer Reported Outcome Measures (ObsROs) was coined and is typically completed by someone other than the patient or a health professional [57].

Traditionally, there has been distinction made between two types of ‘proxy’ response. First, involving someone else (a proxy) responding based on their *own impression* of the individual’s HRQoL (known as ‘proxy-proxy’ [58]); this is consistent with the definition of an ObsRO [57]. Second, a proxy responding based on *how they think* the individual would rate their own HRQoL if they were completing it (i.e., as if they were them, known as ‘proxy-patient’ [58]). Guidance on the use of proxies is not always readily available and varies by instrument [59]. Research also suggests that report by proxy type (i.e., ‘proxy-proxy’ vs. ‘proxy-patient’) may have a differential association with child self-report, where available [60]. For infants and toddlers, it is not theoretically appropriate to advocate a ‘proxy-patient’ perspective, given that the child themselves is not capable of reliably reporting on their HRQoL using a questionnaire [61].

Whatever the definition used, it is recognised that achieving ‘accurate’ responses from a proxy perspective is challenging, and differs depending on the construct being measured, as well as the choice of proxy [62, 63]. Developers of instruments typically take care to reduce subjectivity and bias with item wording, instruction etc. However, subjectivity in reporting is unavoidable and will be influenced by several factors (e.g., experience as a parent/caregiver, experience of ill health, recall, amount of time spent with child etc.). Primary caregivers may differ in their interpretation of items, which may or may not align with the intended definitions established by researchers. This consideration is particularly important in populations with non-dominant language backgrounds or those with lower levels of literacy [64]. High quality cognitive interviewing is essential to identify and help remedy this [51].

The individual serving as the proxy for assessing HRQoL in infants and toddlers could vary (e.g., parent, healthcare professional, childminder). However, one anticipates that the strongest case could be made for the primary caregiver (e.g., the mother, father or other legal guardian) to act as proxy wherever possible. This is a position supported in the latest NICE Methods Guide for HTA [6, 65]. Primary caregivers have the closest and most prolonged interactions with their infants and toddlers and also typically make important health decisions for their child.

#### Proposed recommendation 4

Due to their intimate and close link to their infant/toddler, the primary caregiver should act as the preferred proxy by default. Although this may vary by sociocultural context or study methodology.

#### Proposed recommendation 5

An observer-based approach to proxy report is essential for infants and toddlers (who cannot self-report). This approach is theoretically consistent with an ObsRO whereby the observer should answer *on behalf of the child* (rather than inferring how the child would respond).

### Challenge 4: rapid developmental change

Another challenge for assessing infants and toddlers’ HRQoL is the rapid developmental change that occurs throughout the first few years of life [66]. These changes undoubtedly affect the relevance and/or importance of functioning and the effects of health. The rapid acquisition of developmental skills is not uniform across areas of learning (gross motor, fine motor, communication, social, emotional and cognition) and there is wide variation in age when mastering different skills is considered normal [67, 68]. This not only complicates the references to observable signs, behaviours or events but also makes grouping of ages within a health measure or for analysis more challenging. It is not necessarily pragmatic to have separate PWMs for infants and toddlers of different ages (between 0 and 47 months), which creates additional challenges (see challenge 4: transitioning between instruments). Instead, within agreed age ranges, it is possible to focus on *commonality* when it comes to important aspects of HRQoL for infants and toddlers and how to benchmark this against expected age-related development.

The aim of any HRQoL instrument in infants and toddlers should be to capture aspects of quality of life relevant to *health* and not variations in normal development unrelated to health (e.g., genetics or environment) [28]. Further, HRQoL scores should not change as an artefact of normal developmental change if HRQoL remains the same. These considerations apply both in the selection of aspects for the HRQoL measurement model and how these are operationalised in the resultant instrument (i.e., how they are described and contextualised for the respondent). For the EQ-TIPS version 3.0, dimensions of movement, interacting with others, and play give a list of developmentally appropriate examples to guide parents with their evaluation. Eating or drinking, sleep and managing emotions give examples of aspects related to these functions that can guide completion e.g., sleep suggests falling asleep, staying asleep, amount of sleep. Pain refers to observable signs in the age group: ‘a child in pain may cry non-stop, be restless or unusually still, make a face, moan, or tell you’.

#### Proposed recommendation 6

Consideration needs to be taken when designing items for measuring HRQoL in infants and toddlers to account for rapid developmental change. Ensure items are constructed to best detect changes in HRQoL and not expected changes in development for the intended age range of the measure.

### Challenge 5: transitioning between instruments

Measuring and valuing HRQoL ‘from the cradle to the grave’ is an aspirational goal for members of organisations such as the EuroQol Group [69] and the Patient-Reported Outcomes Measurement Information System (PROMIS) [70]. Doing so, however, raises several conceptual and methodological issues. Any changes in either the way HRQoL is described or measured (including different content or shifting from proxy to self-report) and/or the preferences (value sets) that HRQoL utilities are based on represents a potential discontinuity in assessment over time [71]. This means that HRQoL scores (and QALYs) can alter, not as a function of any genuine change in underlying HRQoL, but as a methodological artefact of the way HRQoL is measured and/or valued. There is thus a tension between developing age-specific HRQoL measures and associated value sets that maximise sensitivity but potentially lack comparability, versus broader age-ranged instruments and/or value sets that maximise the opposite. In general, the more age-based transitions that occur in instruments and/or value sets over the lifespan the greater risk to continuity in measurement over time.

The extent that continuity in HRQoL measurement across the lifespan matters depends on the intended use of the data. For example, it is likely to represent more of an issue where comparisons across or between age groups are necessary. It may also have greater implications in certain applied contexts. For example, recent work is exploring the implications of discontinuity in HRQoL values on QALY estimates in economic models [72]. Furthermore, the extent (and risk) of any discontinuity in measurement will depend on the size of the ‘change’ when transitioning between instruments (e.g., there is greater discontinuity if instruments cover different domains, rather than a rewording of an item that is intended to map onto the same underlying domain of HRQoL).

The issue of transitions in HRQoL measures are more prevalent for assessing child health, and particularly younger children, where greater changes in both the relevance of HRQoL concepts and the way they are assessed may be expected. The PedsQL generic measurement system for example has six different versions across 0–18 years (all designed to map onto the same underlying latent constructs [35]). Other disease-specific paediatric measures have been designed to optimise commonality in assessment (and utility) with the same instrument across a wider age range, such as the DMD-QoL (designed from 7 years of age into adulthood) [73].

In its development, EQ-TIPS prioritised age-related content validity of its dimensions as opposed to continuity with the EQ-5D-Y. Although there is notable overlap between some dimensions of the EQ-5D-Y and EQ-TIPS (e.g., mobility and movement, pain or discomfort and pain) this is not as clear for all dimensions (e.g., usual activities and play or interacting with others). The EQ-TIPS also includes new dimensions not covered by the EQ-5D-Y (e.g., eating or drinking and sleep), considered by caregivers and experts to be relevant to the age group. Further research will be needed to assess the validity of the EQ-TIPS in older ages (e.g., 0–5 years) and/or understand the implications of transitions between descriptive systems (e.g., from EQ-TIPS to EQ-5D-Y) [74].

#### Proposed recommendation 7

Consider the importance of continuity in HRQoL assessment as part of the measurement model and valuation strategy in young children. If the intended context of use prioritises consistency over time (e.g., comparing across/between age groups and/or consistent QALY generation) then develop measures that maximise commonality across age groups. Otherwise, age-based sensitivity could be prioritised (e.g., through age-specific measures).

## Challenge 6: spillover effects

Due to the high level of dependence of infants and toddlers, separating the HRQoL of the child, their caregiver, and potentially wider family represents another major research challenge [28]. Bidirectional spillovers between children and their families in terms of HRQoL outcomes are inevitable, particularly for those living with chronic illnesses, which may increase dependency. The development of the NIHRQOL conceptual model in infants, for example, documents that caregivers found it difficult to discriminate infant HRQoL from their own [28]. If HRQoL measures designed for infants and toddlers (completed by caregivers) capture some of the HRQoL impacts experienced by caregivers or the wider family, which are also captured with complementary assessments, then this can lead to ‘double counting’. This is a practical challenge when these data are used in concert, such as in economic health evaluations [75]. Spillover effects are also possible in the valuation of instruments for PWMs, whereby trading off quality against length of life is difficult without considering the impact on the wider family [13].

The intention of HRQoL measures designed for infants and toddlers is thus to assess the HRQoL of the child, but not the caregiver or family. Family spillover effects are of course important, particularly in the case of health economic evaluations [76], but should be accounted for independently by complementary instruments, where appropriate. Data collection may be challenging in certain contexts, such as with parents caring for very ill children where research participation is especially burdensome, yet can be achieved with appropriate care [77].

The ISPOR task force for Family and Caregiver Health Spillovers in Health Economic Evaluations (SHEER) recently provided several recommendations for good practice in this area [75]. As well as accounting for and flagging the potential for double counting in HRQoL measurement research methodology, the task force recommending using the same measure to assess spillover and patient outcomes where possible to facilitate aggregation in the model. Meeting this recommendation is a clear challenge with infant and toddler HRQoL, where a measure of parental or familial HRQoL is unlikely to look the same as that for the infant or toddler. Nevertheless, the same ‘family’ of measures could be completed, such as the PedsQL variants (including the family impact module) [78] or the EuroQol suite of measures. Alternative methods are proposed if the measures differ, such as conversion algorithms for care-related quality of life to HRQoL outcomes or utilising a cost-consequence analysis [75].

### Proposed recommendation 8

HRQoL measures for infants and toddlers should be designed to minimise capturing ‘spillover’ HRQoL of caregivers or wider family, which can be assessed using complementary, independent instruments.

## Conclusions

Accurate measurement of HRQoL in infants and toddlers is important, yet methodological challenges exist that require pragmatic solutions. Informed by insights as part of the EQ-TIPS project, this commentary outlines six challenges in measuring infant and toddler HRQoL and presents eight proposed recommendations for HRQoL researchers (Table 1). This work should be viewed as complementary and additive to other commentary pieces in the field [5; 13; 56]. We concur with Germain et al. about the need for a clear, consensual core conceptualisation of HRQoL in very young children and about the challenges of proxy report [5]. However, the current paper offers a more expansive and contemporary perspective on the multiplicity of challenges of HRQoL measurement in infants and toddlers, including a set of proposed recommendations. Devlin et al. provide a related article on the challenges of valuing health for infants and toddlers, which can be viewed as complementary when thinking holistically about measuring and valuing HRQoL in very young children for HTA [13].

Our recommendations are limited as they are based on the informed perspectives of one research team and are not intended to be definitive. Instead, they should be viewed as a starting point for further methodological development, debate, and iterative refinement as further work and evidence on the measurement of HRQoL in infants and toddlers is amassed. Neither is it implied that the recommendations proposed here are exhaustive. Other issues such as equity concerns and cross-cultural adaption are likely to be important [56]. For example, instruments designed for infants and toddlers must account for individual differences in ability, culture, and linguistic background, given the increasingly diverse populations of young children for whom these tools are intended. Furthermore, our focus has been on single, questionnaire-based assessments of HRQoL. It could be argued that an effective and comprehensive assessment of infant and toddlers’ HRQoL may require a multimethod approach, combining techniques such as primary caregiver’s reports, direct observations, and interviews.

Ultimately, it is hoped that consensually-agreed and standardised methodological positions can be established to help inform best practice in the measurement and valuation of infants and toddlers’ HRQoL. A consensual and standardised approach is likely to be of benefit both to the research field – by improving comparability and reducing heterogeneity [46] – and to HTA, by facilitating agreed approaches to utility measurement and elicitation in very young children that minimise uncertainty and methodology-induced bias [47].

## Data Availability

No datasets were generated or analysed during the current study.
